# Privacy-Preserving Record Grouping and Consent Management Based on a Public-Private Key Signature Scheme: Theoretical Analysis and Feasibility Study

**DOI:** 10.2196/12300

**Published:** 2019-04-12

**Authors:** Stephan Jonas, Simon Siewert, Cord Spreckelsen

**Affiliations:** 1 Department of Informatics Technical University of Munich Garching Germany; 2 Department of Medical Informatics Uniklinik Rheinisch-Westfälische Technische Hochschule Aachen Aachen Germany

**Keywords:** asymmetric cryptography, public-private key, long-term trials, clinical trials as topic, data anonymization, pseudonymization

## Abstract

**Background:**

Clinical and social trials create evidence that enables medical progress. However, the gathering of personal and patient data requires high security and privacy standards. Direct linking of personal information and medical data is commonly hidden through pseudonymization. While this makes unauthorized access to personal medical data more difficult, a centralized pseudonymization list can still pose a security risk. In addition, medical data linked via pseudonyms can still be used for data-driven reidentification.

**Objective:**

Our objective was to propose a novel approach to pseudonymization based on public-private key cryptography that allows (1) decentralized patient-driven creation and maintenance of pseudonyms, (2) 1-time pseudonymization of each data record, and (3) grouping of patient data records even without knowing the pseudonymization key.

**Methods:**

Based on public-private key cryptography, we set up a signing mechanism for patient data records and detailed the workflows for (1) user registration, (2) user log-in, (3) record storing, and (4) record grouping. We evaluated the proposed mechanism for performance, examined the potential risks based on cryptographic collision, and carried out a threat analysis.

**Results:**

The performance analysis showed that all workflows could be performed with an average runtime of 0.057 to 42.320 ms (user registration), 0.083 to 0.606 ms (record creation), and 0.005 to 0.198 ms (record grouping) depending on the chosen cryptographic tools. We expected no realistic risk of cryptographic collision in the proposed system, and the threat analysis revealed that 3 distinct server systems of the proposed setup had to be compromised to allow access to combined medical data and private data. However, this would still allow only for data-driven deidentification. For a full reidentification, all 3 trial servers and all study participants would have to be compromised. In addition, the approach supports consent management, automatically anonymizes the data after trial closure, and provides basic mechanisms against data forging.

**Conclusions:**

The proposed approach has a high security and privacy level in comparison with traditional centralized pseudonymization approaches and does not require a trusted third party. The only drawback in comparison with central pseudonymization is the directed feedback of accidental findings to individual participants, as this is not possible with a quasi-anonymous storage of patient data.

## Introduction

### Background

Medical progress relies on evidence from trials involving patients, healthy participants, or both. Many relevant study designs (especially cohort studies) require long-term efforts, which need to merge data for each participant. The same holds true for studies that capture and then integrate data from heterogeneous data sources or from different locations to describe individual participants. While handling sensitive health data, such studies require the highest standards of data privacy and data security. Specifically, they have to ensure that sensitive data cannot be traced back to individual participants by unauthorized access. Obviously, these two requirements—enabling record grouping for individual participants and disabling the identification of participants (given the data)—are extremely hard to reconcile even if data are managed by a single institution or study center. Here, data can be collected, then joined using participants’ identifiers, and anonymized only before data analysis. This involves a high risk of leaking identifying information in case of security breaches before anonymization. In addition, anonymization needs to minimize the reidentification risk based on characteristic combinations of superficially anonymized data.

The problem is addressed by establishing *k-anonymity* [1], meaning that the identifying information of a person is indistinguishable from at least k−1 other datasets. Often k-anonymity can only be achieved by generalizing some attribute values, for example by reducing birthdates to ages. Further approaches to solve the reidentification problem involve a more sophisticated protection against attacks due to low data diversity and background knowledge in k-anonymized datasets [2] or small systematically randomized changes to the original data considered irrelevant for subsequent data analytics, but obfuscating individual characteristics [3].

Applying anonymization at the very end of data acquisition is most problematic in long-term studies while sensitive health data are stored nonanonymized over long periods of time, leaving the data vulnerable to attackers. With distributed data acquisition for the same patient, a downstream anonymization prior to data communication between trial centers becomes simply unfeasible, as identifying information needs to be shared to merge patient data.

Let’s assume the following use-case as an example of potential risks. A clinical trial is performed to monitor population depression through weekly Web-based questionnaires. Each data entry (1 filling of the questionnaire) is stored as an individual record, and records are linked through the users’ accounts.

*Immediate* anonymization is not an option here. Simple k-anonymization of the data would result in losing the possibility to link new records to existing datasets and, thereby, participant-individual trend assessment would not be possible. Instead, data might be pseudonymized. Pseudonymization removes all data directly identifying a person (such as name, address, and place and date of birth) and replaces this information with a generated data key, which, considered solely, will not unveil any hint leading to the real person, but is associated consistently with all data describing the same person. Consistent association of the same pseudonym with a person’s data is the main task to be solved by pseudonymization approaches. Solutions established so far either (1) use a pseudonymization table or dictionary, which serves as a lookup device when new data need to be pseudonymized, or (2) adopt a function, deterministically calculating the pseudonym from identifying data [4] (ie, a hash function).

Even enhancing plain pseudonymization with encryption yields some problems. At any point in time, the records of the study participants could be grouped by pseudonym and possibly identifying information could be drawn from the combined information. Even the encryption of pseudonymized data by some master secret poses a risk, as an attacker would need to obtain only a single key.

The optimal solution to the attack scenario stated above would be to fully decouple identifying and study data during acquisition, meaning that no connection between the submitting participant and the records can be drawn at any point in time.

Such a solution needs to (1) remove all directly identifying data and (2) avoid using the same (pseudonym) key for datasets related to the same person. However, (3) records should still be grouped by study participant at the end of the study to analyze individual trends, and (4) participants should be enabled to trigger the deletion of all their data without disclosing their identity.

### Objective

To the best of our knowledge, no such technique is yet available. Therefore, we propose a strong, decentralized pseudonymization technique based on shared public-private key cryptography that has the potential to de facto anonymize study data on acquisition while maintaining the possibility to group data by participant. In the following, we introduce the approach, demonstrate feasibility based on a proof-of-concept implementation, report related performance data, and address scalability based on adopting distributed computing (MapReduce).

## Methods

### Public-Private Key Scheme

To disconnect identifying participant information, such as user account name or email address, from study records, digital signatures based on asymmetric cryptography are employed. Users sign their records using a private key known only to them. These private keys are encrypted by a user’s password and stored on a central server. Each time users add a new record to their dataset, they sign the record using their private key. Thus, each record is stored with a 1-time pseudonym (the signature), which can still be grouped based on available public keys without knowing the private key and, thus, without knowing the participant who created the record.

The matching public keys for each user are centrally stored on the server side. The records can be grouped by verifying the records’ signatures using the combination of all public keys. Wherever possible, cryptographic salt (a random sequence) should be added to hashes and signatures to increase entropy. The salt is stored alongside the hash or signature in the same database. For example, if a user submits the same answers to a questionnaire twice without salt, the same signature is created for both records; thus, they can be grouped without knowledge about the public key. With a random salt, 2 different signatures are created, since the sequences differ and grouping without knowledge of the public key is not possible.

### System Setup

**Figure 1:**
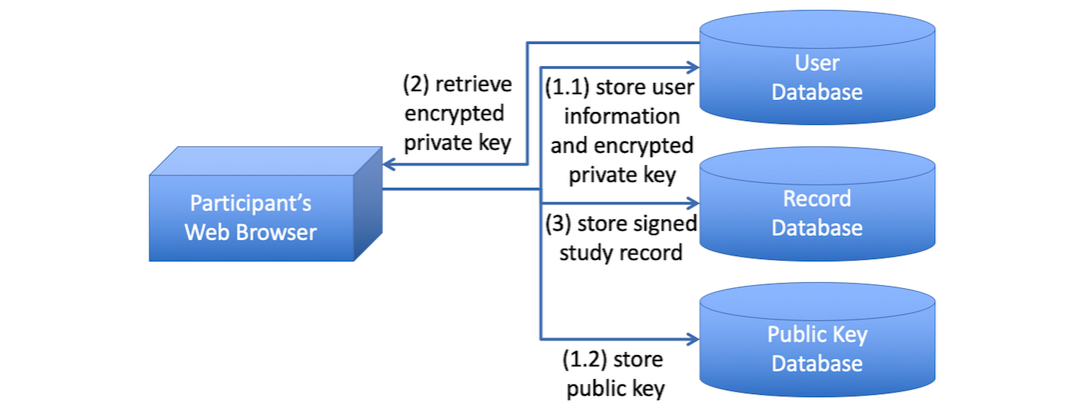
Study database division and workflows.

To ensure further privacy, the user data can be distributed over several servers (Figure 1). Identifying information such as email addresses or user names is stored in a *user database*. Study data such as filled questionnaires are stored in a *record database*, along with the record signature of the user and added cryptographic salt. For cryptographic data, a cryptographic hash with added salt of the user password is stored in the user database for identification; private keys are stored encrypted by the user’s password in the user database with added cryptographic salt; and public keys are stored in a separate *public key database*.

### Workflows

The main workflows can be split into 4 activities: (1) user registration, (2) user log-in, (3) record storing, and (4) record grouping.

During (1) *user registration*, a user with their respective public-private key pair is created. This is performed on the client side. The user chooses a username and password, and a public-private key pair is generated in the Web browser. The following information is then transmitted to the server infrastructure (Figure 1). First, the user database stores the potentially identifying user information such as user name and email address, as well as a cryptographic hash of the user’s password with added cryptographic salt. In addition, the private key is symmetrically encrypted with the password as key and stored in the user database as well. Second, the public key database stores the public key. Both substeps are carried out independently and use separate databases.

On (2) *user log-in*, the user database is queried for the salt, and the hashed user password is generated on the client side and transmitted to the user database for authentication. If the authentication was successful, the encrypted private key is also returned and decrypted using the user’s password in the Web browser.

Once logged in, the user can (3) *generate and store a new record*. The record is treated as binary data and hashed to reduce the amount of data to be signed. A cryptographic salt is added to the hash and the full sequence is signed with the private key. Record, salt, and signature are stored in the record database.

**Figure 2:**
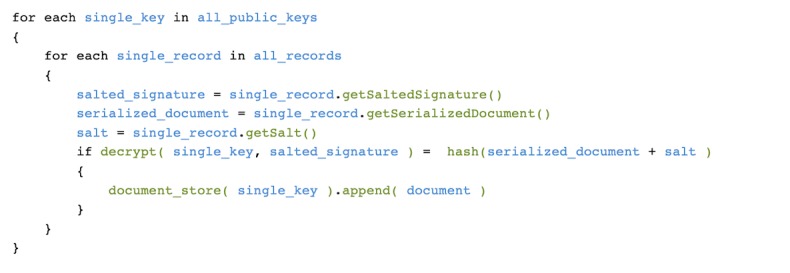
Grouping algorithm as pseudocode.

**Figure 3:**
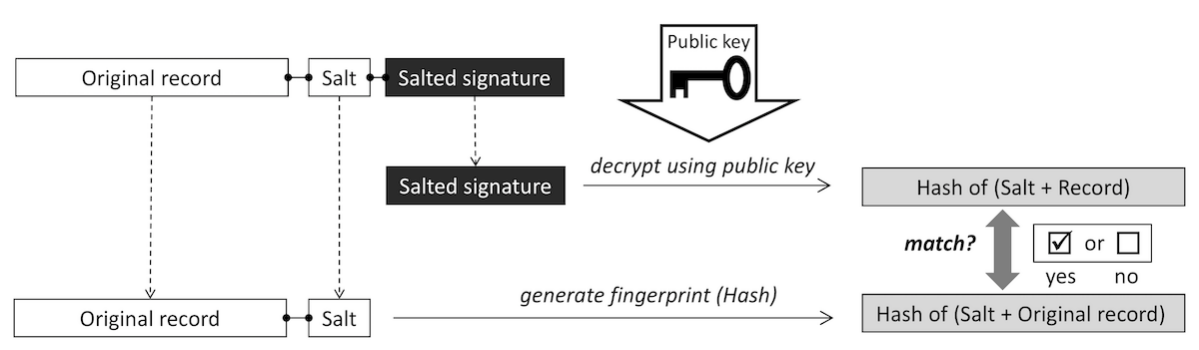
Standard procedure to verify a digitally signed document.

**Figure 4:**
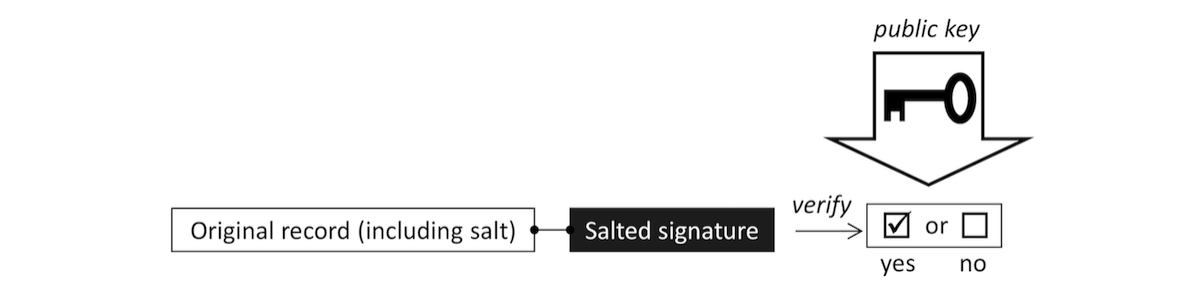
Shorthand graphical notation for the verification step of a digitally signed document as detailed in Figure 3.

The records can be (4) *grouped* using the available public keys from the public key database. For this task, every record and salt from the record database is loaded and the signature is decrypted using each public key. If the decrypted value is identical to the hash of the record and salt, the signature was created with the corresponding private key (Figure 2). This is the well-known standard procedure for verifying a digitally signed document (Figure 3). The procedure is used here to assign all records that can be verified with the same public key to the same group. For the sake of a better visualization of our approach, we introduce a graphical shorthand notation of the verification process in Figure 4.

One crucial point should be highlighted: our approach never discloses the identity of the key owner. This is in sharp contrast to the usual verification of digitally signed documents, which relies on an approved association of the signer’s identity with the public key. Instead, our approach collects the public keys of all participants in a public key store without any trace to the key owner’s identity.

**Figure 5:**
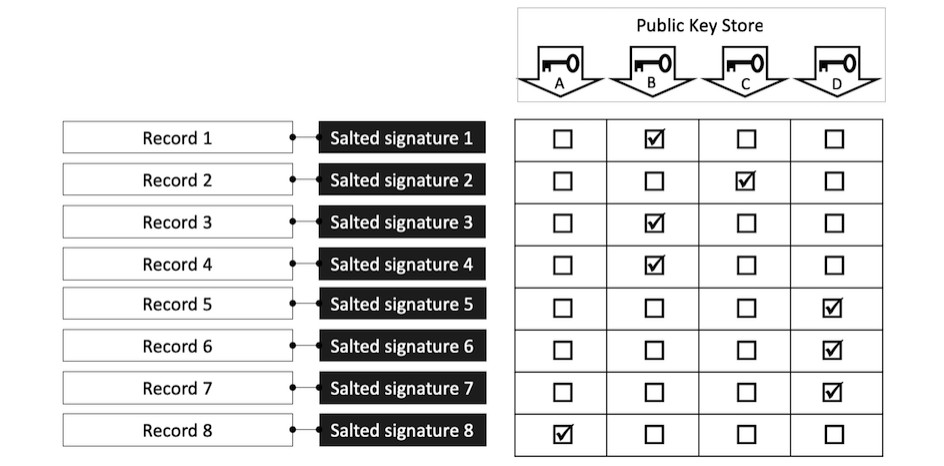
Record grouping using the public key store.

In order to group the records (ie, to join all records belonging to the same, but unknown, participant), all public keys are applied to all records. Records verified by the same public key must have been created using the same private key and, thus, belong to the same participant. Figure 5 shows the essence of this approach.

### Notification to Participants of Trial Analysis

In most trials, participants should be notified if specific diseases or other incidental findings are generated based on the collected data. In our example, a participant could be notified that depression has been diagnosed based on their replies. While our approach does not allow for direct communication to an individual patient identified based on their record, it still allows for directed feedback to just 1 distinct patient. This can be performed by encrypting a message with the public key associated with the diseased participant and sending this encrypted message to all participants (eg, through a mobile app or the Web interface of the trial). Since the message is encrypted with the distinct patient’s public key, only the associated private key is able to decrypt and read the message. Delivery to the correct patient is therefore guaranteed. However, since all patients need to be contacted for the right patient to log in, patients might be confused by potentially alarming messages. Thus, specific times should be scheduled for the communication (or log-in of the patient) or these findings should be communicated with general feedback from the study through the Web interface.

### Public Key–Based Consent Management

While the approach actually relies on the mechanism of digital signatures, it is possible to implement privacy-preserving, participant-managed consent declaration. The participant’s declaration of consent is treated exactly like a record. It is signed electronically by the participant’s private key and enclosed in the record database. While the document is signed digitally, there is no need for further identifying information in the declaration. If all participants sign a declaration of consent, a study center will always be able to prove the righteous use of all personal data. The center only has to show that each record contained in the record database can be grouped with a consent declaration using the quasi-anonymous grouping mechanism introduced above (Figure 5).

**Figure 6:**
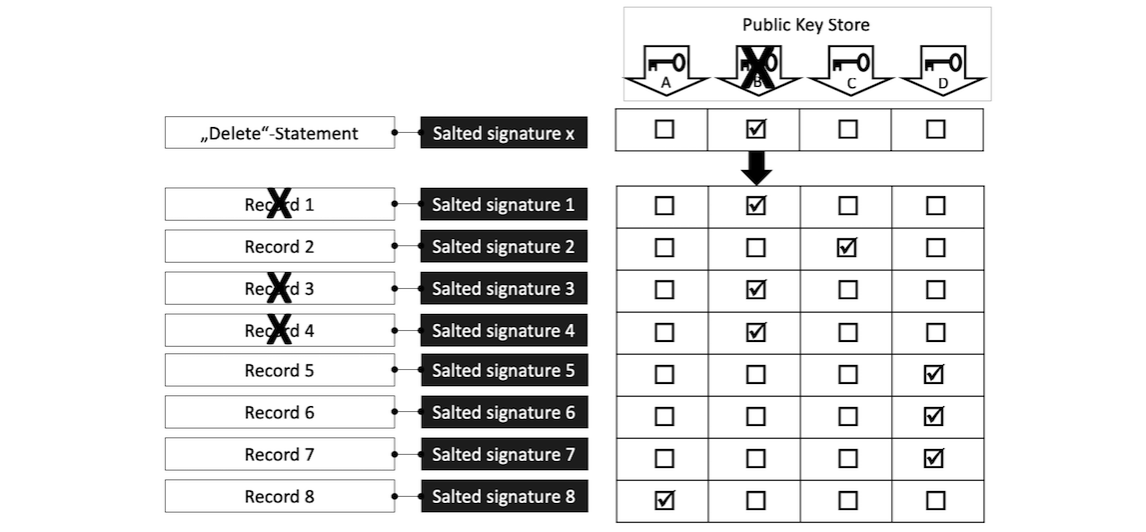
Process for data management after withdrawal of consent.

Using a similar process, a participant can withdraw consent and trigger the deletion of his or her data. The system offers all participants the ability to sign and enter a delete statement. The delete statement is first treated like a usual record and grouped with all records related to the participant (including the consent declaration). Statements associated with a delete statement are flagged for deletion. A garbage collecting mechanism can then clean the record database. Additionally, for any group containing a delete statement and at least one record, the associated public key is also marked for deletion and then removed from the public key store. With these 2 steps, data of participants who withdraw consent are completely removed from the database (Figure 6).

All operations for record grouping and consent management can be implemented as a MapReduce problem and can be addressed using big data technology such as Apache Spark or Hadoop.

Please note again that neither passwords nor private keys are transmitted or stored in clear text to the server at any point in time. Thus, records are grouped and consent is managed quasi-anonymously. In addition, no trusted third party is required for consent management (especially for handling revoked consent).

### System Performance Evaluation

To evaluate the system regarding its computational performance and robustness, we performed several tests. The most expensive operation during user creation is the generation of asymmetric key pairs. Record generation and grouping speed is mostly dependent on the signing time of the record hash. Thus, we calculated runtimes for (1) user registration, (3) record storing, and (4) record grouping using different asymmetric encryption algorithms. In addition, we investigated and calculated possible collisions during calculation of hashes and public-private key pairs. Finally, we analyzed the potential threat of data exposure in various attacker scenarios.

## Results

### Runtime Considerations

We performed all tests using the Bouncy Castle application programming interfaces of the cryptographic algorithms [5]. We focus the report on the main runtime limitation of the asymmetric signing of documents. However, we tested different algorithms for hashing and symmetric encryption but do not report them separately. For the cryptographic hash function, we tested secure hash algorithm (SHA) -1 [6] and SHA-2 [7] with hash lengths of 256 and 512 bits. Since the performance impact on both record generation and the critical grouping operation was negligible, we chose SHA-256 as the hashing function.

We symmetrically encrypted users’ private keys using the Advanced Encryption Standard (AES) [8]. We tested the standards AES-128 and AES-256 with the same result as for hash functions. The performance impact of the used block size did not affect the performance at all. We encrypted the users’ private keys in the following simulations using AES-256.

We tested the following algorithms for the cryptographic signatures: ECGOST3410 (pure elliptic curve), elliptic curve version of the digital signature algorithm (DSA) (ECDSA) [9], SHA-256 DSA [10] (large integer factorization), and SHA-256 Rivest, Shamir, Adleman (RSA) [11] (large integer factorization). We tested all algorithms in a scenario with N=100 users and a mean of 1000 records with a size of 4096 bytes. We repeated each test 5 times and report the average result (Table 1).

**Table 1:** Average timings of common signature schemes for single operations using record length *l*=4096 bytes.

Signature scheme	Runtime (ms)		
	User registration	Record storing	Record grouping
ECGOST3410	0.350	0.342	0.198
SHA-256^a^ DSA^b^ 1024 bit	0.145	0.083	0.067
SHA-256 ECDSA^c^	0.057	0.089	0.011
SHA-256 RSA^d^ 2048 bit	42.320	0.606	0.011
SHA-256 RSA 1024 bit	4.088	0.154	0.005

^a^SHA-256: secure hash algorithm with 256-bit hash length.

^b^DSA: digital signature algorithm.

^c^ECDSA: elliptic curve digital signature algorithm.

^d^RSA: Rivest, Shamir, Adleman.

### Cryptographic Algorithm Collision Considerations

Two potential problems of the presented approach are (1) the chance of collision during signing and (2) the chance of collision during hashing. A collision in signing would yield an incorrect grouping of records. A collision in hashing would allow the injection of false data into the record database.

Given an existing record and its salted signature, a collision during hashing would enable an attacker to find a combination of different salt and record leading to the same hash. The attacker could then assign the preexisting signature to the new pair of salt and record and, thus, induce false but seemingly valid data. Since SHA-256 is used for hash computation, the hash length is 256 bits. The probability of a collision (P_col_) can be approximated by applying the *birthday problem* [12] in the equation P_col_(*m*, *o*) = 1 – *e* [– *m* (*m* –1)/2^*o*^^+1^], where *m* denotes the number of already computed hashes (record count *m*) and *o* is the output size of the hash function in bits. For the practical simulation, one therefore gets a collision probability of P_col_(105,256) ≈ 0. Actually, the probability of observing at least a single collision reaches 50% for *m* ≈ 4 × 10^38^ hash operations. Thus, introducing false (and, more importantly, specific) data into the record database or having 2 records by the same person with the same hash is near impossible.

In the first case (signing), a collision can occur if 2 public keys are able to decrypt the same signature. Since the signing process is bijective, this means the 2 public keys have to be identical. However, the key length is even larger for the public keys than for the hashing algorithms; thus, the probability is even lower and poses no likely threat.

In contrast, the signing mechanism can almost guarantee the validity of the data, making forging (except for deletions) almost impossible.

### Threat Analysis

Due to the layout of the system, the data are highly scattered between several (physically separate) database systems. We assume a retrospective attack after data have been entered for the following cases.

In the case where 1 database is compromised, that is an attacker gains access to any single database, it will not be possible to link a single participant to any of their study data, as none of the databases contains any connection between identifying data and records or between study and identifying data at the same time.

In the case where 2 databases are compromised, if the attacker gains access to the user and public key databases, they would not have access to study data. If the attacker gains access to the user and record databases, they would have no means of linking data from the 2 databases or even within the record database. If the attacker gains access to the record and public key databases, they would potentially be able to group records of the same user but would not have access to identifying information.

In the case where all 3 databases are compromised, the attacker would still only be able to group the records together if they know the cryptographic methods that were used. In this case, they would still have no connection between study data and identifying data, but they might be able to identify study participants based on behaviors or answers in the records.

In the worst case, to completely decrypt and link every single person with their study data (matching user name or email address with records), an attacker would have to gain access to each individual participant’s computer and record the private key or password during the user log-in procedure, as well as gaining access to the records database and possibly the user database. This is not possible retrospectively, as the study is finished and no log-ins are made anymore.

## Discussion

### Principal Results

We present a cryptographic scheme for decentralized pseudonymization, participant information, and participant-managed consent declaration and withdrawal. The technical evaluation of runtime and collisions indicated not only that such a system is feasible, but also that runtimes are short enough to be integrated without notification or impairment of user experience. Especially when the focus is on small computational expenses on the user’s end, the SHA-256 ECDSA is a good choice for signature generation, as it provides high security with a short runtime. SHA-256 ECDSA is also used in other cryptographic online tools such as Bitcoin. The chosen approach of signing each patient record individually also eliminates the risk of accidental or willful data tampering (except for record deletion).

This approach can be classified as decentralized pseudonymization, with the private key being part of the pseudonymization function. However, since the study personnel have no access to parts of the pseudonymization procedure and cannot interfere with it, the approach may potentially even be classified as de facto anonymization. If participants withdraw their consent, no depseudonymization is necessary. Records are marked for deletion and removed by a garbage collection mechanism without disclosing a participant’s identity. Obviously, cooccurrence analysis of user interaction and deletion operations could leak identifying information. This risk can be minimized by (1) strictly separating the systems for user and record management, (2) cumulating record deletion requests for 2 or more users, and (3) avoiding a detailed log of user interactions.

The threat analysis showed that major effort would be needed to link identifying information with study data. Theoretically, gathering identifying personal information is only possible during the study and not after a study has been completed. A closed study might therefore be automatically anonymized. However, de facto security is highly dependent on implementation details, and—as history has taught us—even standard software libraries are prone to errors.

### Limitations

The main limitation of the proposed method is that the user’s private key becomes unrecoverable if the user forgets his or her encryption password. Without decrypting the private key, a user is not able to insert new records into the database to be grouped with older records. There are multiple possibilities available to reduce the risk of losing a key. One option would be to hand out the users’ private keys in the form of smartcards. Losing a physical object is much less likely than forgetting a password. Another approach would be to store a copy of the private key encrypted with a secret obtained from the answers to a set of user-selected questions. Both approaches would decrease the risk of losing a private key but not totally eliminate it. In addition, these approaches might not be feasible in all settings or for all participants, which creates the risk of losing a study participant’s data.

Similarly, since the trial is theoretically anonymized once the last participant has finished data entry, there is only an indirect way of communicating with study participants: through broad messaging to all participants. This might also not be feasible or successful in all cases. This limitation should be communicated to participants beforehand. A potential solution to this problem could be the addition of a trusted third party for those participants who value a direct follow-up more than anonymization of their data. Alternatively, an accompanying mobile phone app could hold the private key and could filter incoming messages and alert the user in case of information relevant to them.

Another limitation is possible collusion during cryptographic hashing. Our analysis showed that this is only a theoretical problem, but it may not be prevented completely.

Access control in general, and specifically the limitations of this approach, might discourage study participants from participating in a trial. However, accompanying mobile phone or tablet apps could reduce this burden by offering more convenient ways for password and key management (eg, face or fingerprint identifiers). However, this could in turn reduce security if private keys are stored nonencrypted on the device over longer periods of time.

### Comparison With Prior Work

Prior work proposed the assignment of identical pseudonyms to records linked to the same participant or patient in order to enable record grouping or patient-specific data joins [4,13]. Pseudonymization is often combined with encryption to ensure both deidentification and confidentiality of the data [13]. Pseudonyms establish linked records within the database during the whole lifecycle of the database. Joining records by pseudonyms yields rich datasets per individual participant, which are, therefore, exposed to a greater risk of information-driven reidentification. In contrast, our approach avoids identical pseudonyms. Record groups are established on demand by the public key store. Thus, based on our approach, the public key store could be kept by a different organizational unit. Record grouping is then postponed to the time of data analysis, which reduces the reidentification risk during the data acquisition phase (especially the high risk due to low data diversity at the beginning of data acquisition; see Machanavajjhala et al [2]).

A main advantage of our approach is that no trusted third party is required for consent management. With respect to this point, our approach differs from an approach proposed by Aamot et al, which also adopted asymmetric encryption [14]: our approach completely avoids depseudonymization, but nonetheless enables withdrawal of consent and patient information.

Noumeir et al made the distinction between reversible and 1-way pseudonymization [15]. They argued that 1-way pseudonymization cannot support any notification of participants about incidental findings. They, therefore, proposed a symmetric encryption of data enabling reidentification by a trusted third party. Our approach is 1-way in the sense that a feedback on incidental findings cannot be propagated back to a distinct participant directly. However, through encryption and broad communication, a similar effect can be achieved. The only drawback is that it requires more activity by the user (ie, actively logging in to the system), which is not necessary in other cases.

### Conclusions

We have proposed a novel cryptographic approach to pseudonymization that decentralizes the pseudonymization function and consent management in part to the study participants. Closed trials are thereby automatically anonymized, and a potential de facto anonymization at the study site during ongoing trials might be achieved. However, this claim requires further investigation and might be dependent on local privacy regulations. A prototypical implementation of the key cryptographic mechanisms of the trial software with grouping based on Apache Spark is available online [16].

## Abbreviations

AES: Advanced Encryption Standard
DSA: digital signature algorithm
ECDSA: elliptic curve digital signature algorithm
RSA: Rivest, Shamir, Adleman
SHA: secure hash algorithm
